# Stem cell enriched-epithelial spheroid cultures for rapidly assaying small intestinal radioprotectors and radiosensitizers *in vitro*

**DOI:** 10.1038/s41598-018-33747-7

**Published:** 2018-10-18

**Authors:** Marimar de la Cruz Bonilla, Kristina M. Stemler, Cullen M. Taniguchi, Helen Piwnica-Worms

**Affiliations:** 1 Department of Experimental Radiation Oncology, The University of Texas MD Anderson Cancer Center, Houston, TX 77030 USA; 2 Department of Radiation Oncology, The University of Texas MD Anderson Cancer Center, Houston, TX 77030 USA

## Abstract

Radiation therapy is one of the main treatment options for many cancer patients. Although high doses of radiation may maximize tumor cell killing, dose escalation is limited by toxicity to neighboring normal tissues. This limitation applies particularly to the small intestine, the second most radiosensitive organ in the body. Identifying small intestinal (SI) radioprotectors could enable dose escalation in the treatment of abdominopelvic malignancies. However, the only assay currently available to identify effects of radiomodulating drugs on the regenerating capacity of SI stem cells is the Withers-Elkind microcolony assay, which requires large numbers of mice, making it a costly and low throughput method. Here, we describe a novel spheroid formation assay (SFA) that utilizes SI stem cell-enriched three-dimensional epithelial spheroid cultures to identify gastrointestinal radiomodulators *ex vivo*. The SFA is scalable for high throughput screening and can be used to identify both radioprotectors and radiosensitizers.

## Introduction

Gastrointestinal (GI) toxicity often limits the amount of chemotherapy and radiation that can be given to cancer patients. This issue is highlighted with radiation therapy, which can kill solid tumors anywhere in the body but also damages adjacent normal tissue. For instance, cancers of the abdomen and pelvis, such as pancreatic and prostate adenocarcinoma, are difficult to ablate with radiation alone because these tumors require high doses of radiation for control, but are often adjacent to very radiosensitive structures of the GI tract, such as the small intestine^1^. This potential for morbid toxicity, often prevents these tumors from receiving a definitive therapeutic dose. In addition, chemotherapeutic agents, such as irinotecan and anti-angiogenic biologics such as bevacizumab, also have dose limiting GI toxicities, including potentially fatal diarrhea and bowel perforation^2,3^.

Despite the fact that toxicity to the GI tract is a significant clinical problem, there are no existing approved normal tissue protectors that can prevent this toxicity. Amifostine is the only FDA approved radiation protector, but its use is limited by severe side effects such as hypotension and nausea^4^. Thus, identification of novel radioprotectors could not only improve patients’ quality of life by reducing the aforementioned side effects, but also increase the therapeutic window to enable dose escalation of cytotoxic therapy and more effective tumor cell killing. The classical assay to assess the cellular radiation response is the clonogenic assay, or colony formation assay, which monitors the ability of a single tissue culture cell to grow into a colony after administration of radiation or chemotherapy^5^. Unfortunately, the discovery of novel protective drugs by this assay is limited by the technical difficulties of culturing normal tissues *ex vivo*. Cell lines such as Caco-2 (colon cancer derived cells) or Hs 1.Int (non-epithelial intestinal derived cells) have been used to approximate intestinal function, but are often inadequate for estimating the ability to respond to radiation since neither of them are truly representative of normal cells^6^.

The gold standard for studying the response of the intestinal tract to cytotoxic insult is the microcolony assay developed by Withers and Elkind^7^, which assesses regenerating intestinal stem cells after cytotoxic therapy. This method utilizes a single lethal or sublethal administration of cytotoxic therapy (e.g. radiation) that kills existing intestinal stem cells in the crypt. Nascent stem cells that regenerate after the cytotoxic insult are identified by a histopathological technique that requires transverse sections of intestine on a single slide, which can be technically demanding^7^. Even if the slides are prepared properly, the individual regenerating crypts must be counted manually by an experienced pathologist. Lastly, implicit in this assay is that each data point requires large numbers of mice, making the entire process both costly and low throughput for identifying and characterizing new modulators of intestinal damage.

The advent of intestinal stem cell enriched-spheroids grown from murine or human tissue *ex vivo* has enabled the study of normal intestinal tissue and its responses to radiation^8^ or chemotherapy^9^. These cultures, which are amenable to genetic modification^10^, can be passaged indefinitely using growth factor-enriched media in a 3D matrix. In this study, we describe the development of a modified colony formation assay, which we refer to as a spheroid formation assay (SFA), that establishes a small intestinal (SI) stem cell-enriched spheroid cell line directly from mice to study the effects of radiation or chemotherapy treatments *ex vivo*.

## Material and Methods

### Study approval

This study was carried out in accordance with the recommendations in the Guide for the Care and Use of Laboratory Animals from the National Institutes of Health (NIH) Institutional Animal Care and Use Committee (IACUC). The protocol was approved by the IACUC at MD Anderson Cancer Center (Protocol #1101RN01). Animals were euthanized as dictated by the Association for Assessment and Accreditation of Laboratory Animal Care International and IACUC euthanasia endpoints.

### Statistical Analysis

The statistical analyses used in this study are described in each figure legend.

### Reagents, media and drugs

Polybrene (Sigma-Aldrich), 1X Dulbecco’s calcium and magnesium free phosphate buffered saline (DPBS) (Corning); Corning® Cell Recovery Solution CS100 ml (Corning); TrypLE™ Express Enzyme (1X), phenol red (Gibco); DMEM/F12 (Sigma Chemical Co.); ADMEM/F12 (Sigma Chemical Co.); HBSS (Ca2+− and Mg2+− free; Life Technologies); Fetal Bovine Serum – Premium Select (Atlanta Biologicals); L-Glutamine, 200 mM (Sigma-Aldrich); Penicillin, 10,000 Units/ml and streptomycin, 10,000 ug/ml (Hyclone); ViaStain™ AO/PI Staining Solution (Nexcelom); Corning® Matrigel® Basement Membrane Matrix, *LDEV-Free, 10 mL (Corning); L-WRN cell 50% conditioned media (prepared as described in^10^); ROCK Inhibitor (Y-27632) (Sigma-Aldrich); TGF-β RI Kinase Inhibitor VI (SB431542) (EMDMillipore); D-Luciferin, Potassium Salt (Proven and Published™) (Gold Biotechnology); N-acetylcysteine amide (NAC), (Sigma Aldrich); WR-1065 (Sigma-Aldrich), SN-38 (Selleck Chemicals); and Dimethyl sulfoxide, Hybri-Max™, sterile-filtered, BioReagent (Sigma-Aldrich).

### Equipment and consumables

The following equipment and consumables were used to develop the SFA: Cellometer® Vision Cell Profiler CBA (Automated Cell Counter); CLARIOstar (BMG Labtech); Cytation 3 (Biotek); Clear cell culture plate, 24 well (Thermo-Fisher); Black wall clear bottom cell culture assay plate, 24 well (MIDSCI); 5 ml Falcon® round bottom tubes with cell strainer cap (Corning); Cellometer® Disposable Counting Chambers (Nexcelcom); X-RAD 320 Biological Irradiator (Precision X-Ray), VX-2500 Multi-Tube Vortexer (VWR).

### Mice

Nine to twelve-week old male and female C57BL/6J mice used to isolate small intestinal crypts were purchased from Jackson Laboratories (stock no. 000664).

### Generation of SI stem cell enriched spheroid cell line

Crypt isolation and establishment of spheroid cultures were performed as described by Miyoshi *et al*.^10^. For all steps in this procedure, DPBS and HBSS without calcium and magnesium were prepared and EDTA was added to a final concentration of 2 mM. All solutions were prepared before initiating harvest and placed on ice. Mice were euthanized and their entire small intestinal (SI) tract was isolated and flushed with DPBS. The SI were cut longitudinally and placed on ice in DPBS for 10 min, then transferred to ice-cold HBSS. Sections were vortexed at 1,600 rpm in HBSS for 5 min, changed to fresh HBSS, vortexed at 1,600 rpm for 3 min, then again placed into HBSS before vortexing at 1,600 rpm for 8 min. The cells were then changed to fresh HBSS and vortexed at 1,600 rpm for 5 min. All vortexing was conducted at 4 °C. Supernatants from the third and fourth vortexes were combined and passed through 70-μm strainers (Corning) to isolate crypts and remove any villi that might remain in the washes. Crypts were pelleted at 100 × g at 4 °C, then resuspended in ADMEM/F12 supplemented with 10% (vol/vol) FBS, 10 U/mL penicillin, 10 μg/mL streptomycin, and 2 mM L-glutamine, centrifuged at 400 × g at 4 °C and resuspended in Matrigel. Crypts were plated in a 24-well tissue culture dish (30 ul per well). After Matrigel solidification at 37 °C, 50% (vol/vol) L-WRN conditioned media was supplemented with 10 μM Y27632 (ROCK inhibitor) and 10 μM SB431542 (TGF-β RI Kinase Inhibitor VI) and added to each culture well. Media was changed every second day and spheroids were passaged every third day.

### Generation of SI stem cell enriched spheroids expressing mCherry and CBR-Luc

Lentiviral transduction of SI stem cell enriched spheroids was carried out as described^10^. Briefly, cells were transduced with lentivirus encoding Click Beetle Red Luciferase (CBR-luc) and mCherry (FUW-CBR-luc-mCherry)^11^ in the presence of 1 μg/ml Polybrene for 6 h.

Cells were expanded for 7 passages, harvested using Cell Recovery Solution, digested to a single cell suspension using TrypLE supplemented with 10 μM Y27632 and 500 μM NAC and mCherry positive cells were isolated using an Influx cell sorter (BD Biosciences). Gating strategy included sorting on first forward/side scatter, then on singlets, and lastly mCherry positive expression. Sytox blue was used to exclude dead cells. Non-transduced cells from Passage 7 parental spheroid cultures served as a negative control for gating in the mCherry channel^11^.

### Spheroid Formation Assay (SFA)

Spheroid cultures were placed in Cell Recovery Solution for 30 min on ice. Samples were transferred to a conical tube, centrifuged at 233 × g for 5 min at 4 °C, washed with DPBS, centrifuged at 233 x g for 5 min at 4 °C, trypsinized using TrypLE supplemented with 10 μM Y27632 and 500 μM NAC for 5 min at 37 °C on a bead bath. Samples were pipetted up and down for 10 s once every minute for 5 min. TrypLE was neutralized with ADMEM/F12 supplemented with 10% (vol/vol) FBS, 10 U/mL penicillin, 10 μg/mL streptomycin, 2 mM L-glutamine, 10 μM Y27632 and 10 μM SB431542. Samples were centrifuged at 233 x g for 5 min at 4 °C, resuspended in ADMEM/F12 supplemented as described above, and placed in a 5 ml Falcon® round bottom tube with 35 μM cell strainer cap to isolate single cells. Live cells were quantified using ViaStain™ AO/PI Staining Solution in the Cellometer® Vision CBA Image Cytometer. Five thousand live cells suspended in 30 μl of Matrigel were then placed in each well of five 24-well culture plates. After solidification of Matrigel at 37 °C, 500 μl of 50% (vol/vol) L-WRN conditioned media containing 10 μM Y27632 and 10 μM SB431542 was added to each well. Media was changed every three days. When spheroids formed (3–8 days), cultures were exposed to the indicated pre-radiation treatment and then irradiated using the X-Rad 320.

### Brightfield SFA

Cells were harvested and passaged as described above, incubated on ice with Cell Recovery Solution for 30 min and transferred to 15 mL conical tubes. They were then centrifuged at 233 x g for five minutes at 4 °C and digested to single cells using supplemented TrypLE. Live cells were quantified and 5,000 live cells were plated in 30 μL of Matrigel per well. Each sample was plated in triplicate on black wall clear bottom cell culture plates. Once Matrigel solidified, samples were supplemented with 50% (vol/vol) L-WRN conditioned media and spheroids were allowed to grow for 5 days. Conditioned media was changed every 3 days. Using a Cytation 3 Cell Imaging Multi-Mode Reader, spheroids were visualized using Z-stack images spanning 1.5 mm in height and covering the entire Matrigel dome area. The imaging protocol acquired 16–25 slices spanning 1500 μm sample depth beginning at the bottom of the culture well and proceeding to the top of the Matrigel dome. Slices were merged to produce a single Z-stack image spanning the entire depth of the Matrigel dome. Twenty-one Z-stacked images were then manually stitched together using Adobe® Photoshop® to generate an image of the entire spheroid culture. Spheres measuring at least 150 μm were manually quantified and surviving fraction was calculated as described previously^5^.

### Bioluminescent SFA

Immediately following brightfield image acquisition, D-Luciferin was dissolved in 50% conditioned media supplemented with 10 μM Y27632 and 10 μM SB431542 to a final concentration of 300 μg/mL. Conditioned media was aspirated from each well and replaced with 500 μl of D-Luciferin-containing conditioned media. Cultures were placed in the incubator for 10 to 30 min at 37 °C. Bioluminescence was measured from the entire well using a CLARIOstar plate reader using top optic with a focal height of 5 mm at 1 second per well with no emission filter.

### Immunofluorescent staining of spheroids

Spheroid cultures were washed with PBS and fixed with 4% paraformaldehyde for 1 h. Cells were collected and resuspended in Histogel^TM^. Tissues were sectioned at a thickness of 5 μm. Triology (CellMarque) was used for deparafinization, rehydration, and antigen retrieval according to the manufacturer’s protocol. Sections were blocked using protein block (Dako). Following blocking, sections were incubated at 4 °C overnight with primary antibody diluted in antibody diluent (Dako). Primary antibody concentrations were as follows: antiphospho-Histone H2AX 1:250 (Cell Signaling, 9718), and anticleaved caspase 3 1:300 (Cell Signaling, 9661). Sections were washed and then incubated with secondary antibody for 30 min at room temperature in the same antibody diluent (Dako). Secondary antibody used was Alexa-Fluor 488 donkey anti-rabbit 1:500 (ThermoFisher, A-21206). Sections for immunofluorescence were washed with PBS, counterstained with DAPI (1 μg/mL) in PBS, washed, and coverslip-mounted using fluorescent mounting media (Dako). Fluorescent images were acquired using a Nikon Eclipse Ni-E microscope, with Nikon Plan Fluor 40x/1.30 objective, Andos Zyla sCMOS camera, and NISElements Advanced Research software.

## Results

### Development of the Spheroid Formation Assay (SFA)

**Figure 1:**
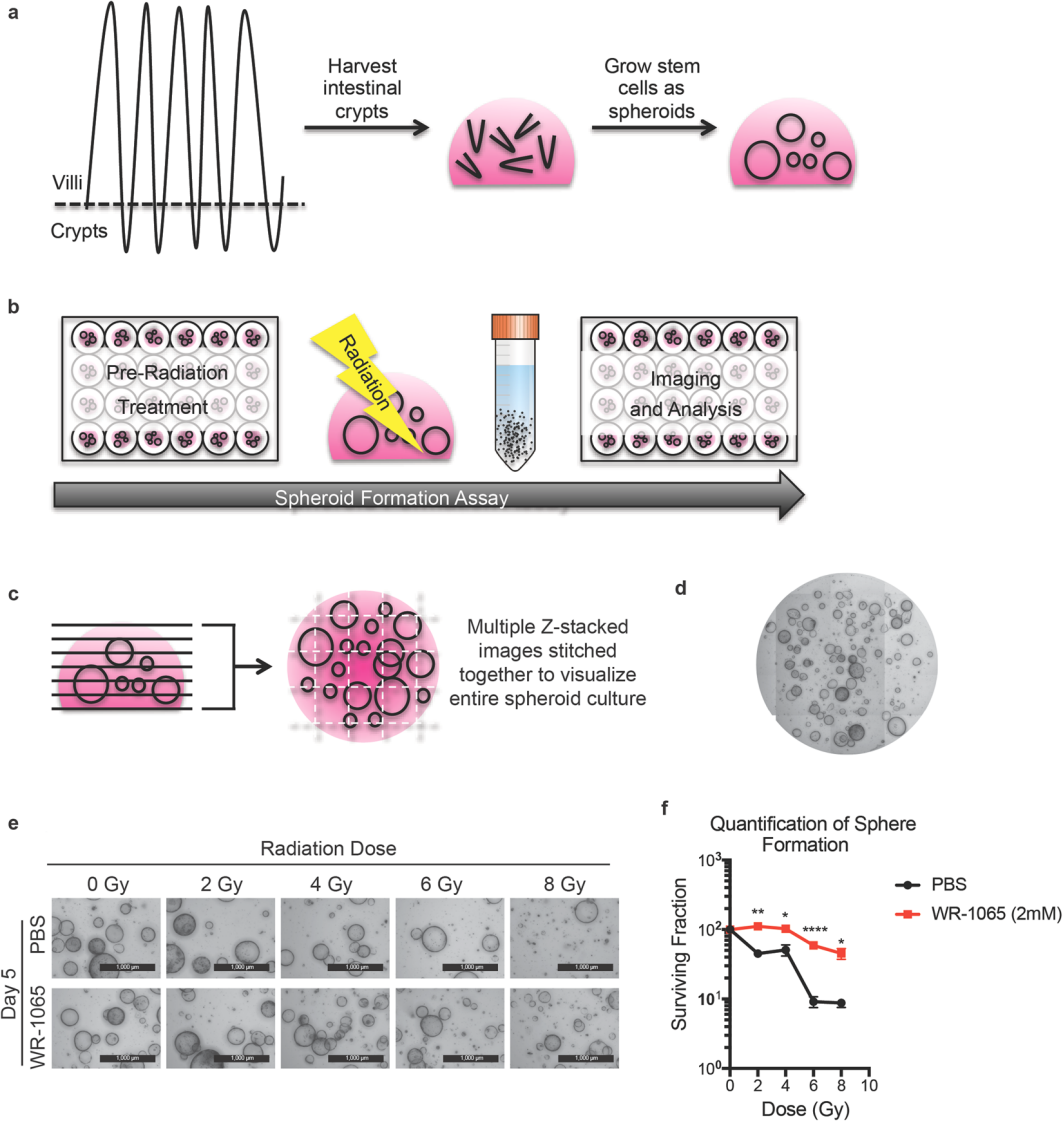
Spheroid formation assay to identify GI radioprotectors. Schematic representation illustrating how stem cell-enriched epithelial spheroid cultures are established (panel a). Schematic outlining how the spheroid formation assay (SFA) is performed (see text for details, panel b). Z-stack images of treated stem cell-enriched epithelial spheroid cultures are stitched together to visualize spheroids (panel c). A representative image of the stitched z-stack is shown (panel d). Stem cell-enriched epithelial spheroid cultures treated with vehicle (PBS) or 2 mM WR-1065 for 2 h were exposed to the indicated doses of ionizing radiation. Spheroids were immediately dissociated into single cells, replated in Matrigel and imaged 5 days later (panel e) (Scale bars, 1000 μm). Spheroids larger than 150 μm in diameter were quantified and mean surviving fraction is plotted. *P < 0.05, **P < 0.01, ***P < 0.001, ****P < 0.0001 by two-tailed, Student’s t test (N = 3 per group). Error bars are ± SEM.

Epithelial spheroid cultures were generated from small intestinal (SI) crypts of C57BL/6J mice (Fig. 1a). Protocols for isolating and culturing stem cell-enriched epithelial spheroid cultures have been described^10^. Briefly, mice were euthanized according to an approved animal protocol, and their SI crypts isolated and embedded in basement membrane matrix (Matrigel). Cultures were incubated at 37 °C for 2 to 4 days to allow primary spheroids to form. Primary spheroids were passaged at least 10 times at 3 day intervals which allowed for the elimination of mesenchymal cell contaminants, enrichment and expansion of non-budding stem cell enriched spheroids, and provision of enough starting material for subsequent experiments. To perform the SFA, spheroids were subjected to the desired experimental treatment, irradiated at the specified doses, isolated from the Matrigel, digested to single cell suspensions, re-plated at a pre-determined concentration, and cultured to re-establish spheroids (Fig. 1b). Spheroid formation capacity was monitored as a function of time.

### SFA assay for identifying radioprotectors

As a proof of concept, spheroid cultures were treated with a known radioprotector to test if the SFA would indeed reflect protection of spheroids after irradiation (i.e. increased spheroid formation after irradiation versus unprotected controls). Amifostine is a validated radioprotector and serves as a prodrug that is actively dephosphorylated to generate WR-1065, the active metabolite of amifostine^4^. Amifostine has been documented as an intestinal radioprotector when administered to mice 30 min prior to radiation. These observations were made using the standard microcolony assay described earlier^12^. This data makes WR-1065 an ideal candidate to test in our assay. Cells were plated at a density of 5,000 cells per well and allowed to grow for 5 days to generate spheroids. Cultures were incubated in the presence of either vehicle (PBS) or 2 mM WR-1065 for 2 h^13^ and then exposed to increasing doses of ionizing radiation. Spheroids were immediately digested to single cells, replated at 5,000 cells per well embedded in a Matrigel dome, allowed to grow for 5 days, and then imaged.

Viable spheroids were quantified using Z-stack images spanning 1.5 mm in height and covering the entire Matrigel dome area (Fig. 1c). Images were manually stitched together (Fig. 1c) and a representative image of the matrigel dome is shown in Fig. 1d. Vehicle-treated (PBS) cultures exhibited reduced spheroid formation capacity with increasing doses of radiation, with very few spheroids visualized at the 8 Gy dose (Fig. 1e). WR-1065 afforded significant radioprotection, even at doses as high as 8 Gy (Fig. 1f). Thus, the SFA successfully replicated a colony formation assay in a 3D culture model.

### Development of modified SFA that employs bioluminescence

**Figure 2:**
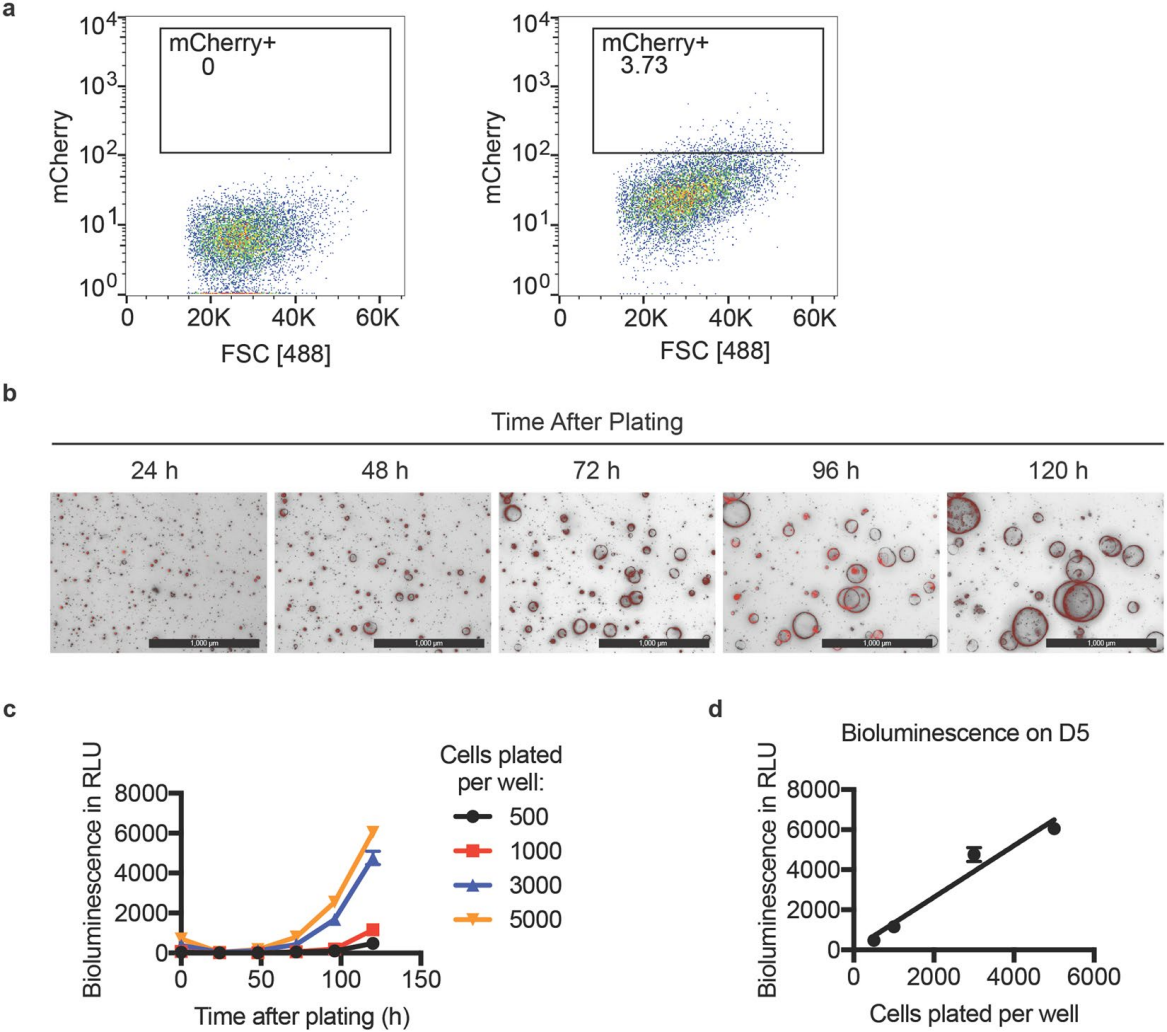
Generation of stem cell-enriched epithelial spheroid line stably expressing both Click Beetle Red Luciferase (CBR-Luc) and mCherry. Cells were transduced with FUW-CBR-luc-mCherry lentivirus and mCherry positive cells were isolated by flow cytometry (panel a). mCherry positive cells were plated and imaged at various times post plating. Representative bright field and mCherry (Texas Red) merged images are shown. Scale bars, 1000 μm (Panel b). Varying amounts of transduced cells were plated and bioluminescence was measured at the day of plating (day 0) and daily until day 5. Bioluminescence obtained for each sample was normalized to the bioluminescence measured in wells containing culture media and Matrigel but lacking cells (panel c). Cells plated per well at day 0 are plotted against the normalized bioluminescence measured per well on day 5 (panel d). P < 0.05 by Pearson correlation (N = 3 per group). Error bars are ± SEM.

To improve the throughput capabilities and quantitation of the assay, we engineered our stem cell-enriched epithelial spheroid line to stably express both Click Beetle Red Luciferase (CBR-Luc) and mCherry using lentiviral transduction (Fig. 2a)^11^. In this way, mCherry expression could be used to select infected cells, while bioluminescence could be used to detect live cells as a function of time. Transduced cells were subjected to fluorescence-activated cell sorting (FACS) to isolate mCherry-positive cells (Fig. 2a), which were then plated at a density of 5,000 cells per well. As seen in Fig. 2b, transduced cells generated mCherry positive, stem cell-enriched epithelial spheroid cultures. Spheres were discernible as early as 48 h after plating a single cell suspension and were still viable 120 h after plating. Single cells were plated in triplicate at different densities and allowed to form spheroids for 5 days. Media containing D-luciferin was introduced into each well of the tissue culture plate and bioluminescence was measured in each well as a function of time: measurements were obtained at the time of plating (day 0) and daily from day 1 through 5 post plating. As shown in Fig. 2c, bioluminescence increased with time, indicating growth of cells. Bioluminescence measured on day 5 positively correlated with the number of cells plated in each well (Fig. 2d, r = 0.9772, R^2^ = 0.955) demonstrating that bioluminescence could be used to monitor the growth of cells in 3D spheroid cultures.

### Bioluminescent SFA to assay radioprotection

**Figure 3:**
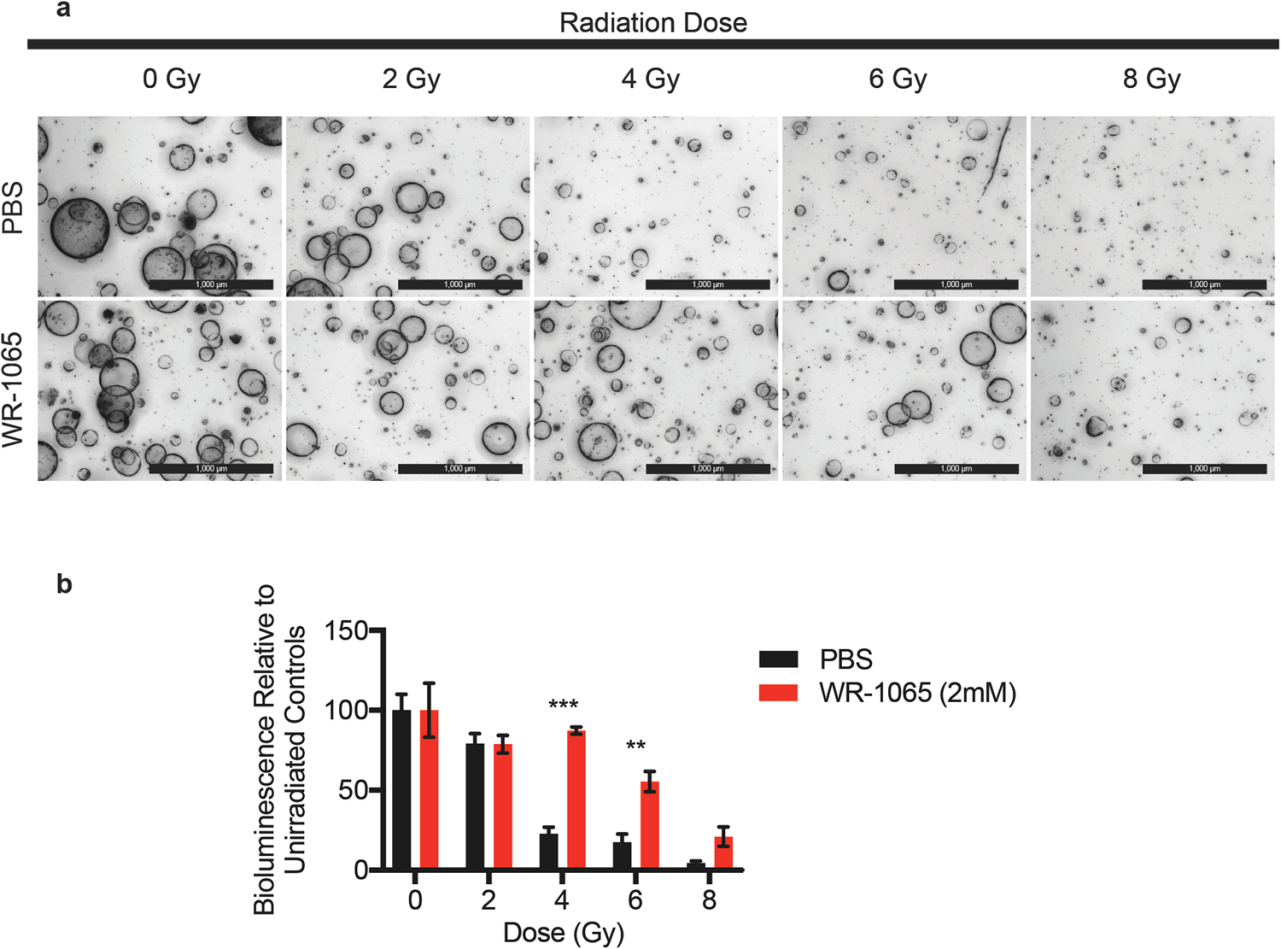
Spheroid Formation Assay for identifying radioprotectors *in vitro*. Stem cell-enriched epithelial spheroid cultures were incubated in the presence of vehicle or 2 mM WR-1065 for 2 h and then either mock irradiated or exposed to increasing doses of ionizing radiation. Spheroids were immediately dissociated to single cells and 3,000 cells were plated per well in triplicate and cultured for 5 days. Representative bright field images (panel a) (Scale bars, 1000 μm) and corresponding bioluminescence measurements (panel b) are shown for day 5 samples. **P < 0.01, ***P < 0.001 by two-tailed, Student’s t test (N = 3 per group). Error bars are ± SEM.

The ability of the bioluminescent SFA to demonstrate WR-1065-mediated radioprotection in spheroid cultures was tested. Single cells were plated at a density of 5,000 cells per well and cultured for 5 days. Stem cell enriched-epithelial spheroid cultures were incubated in the presence of PBS (vehicle) or 2 mM WR-1065 for 2 h and were then either mock-irradiated or exposed to increasing doses of ionizing radiation. Spheroids were immediately dissociated to single cells, after which 3,000 cells were plated per well and cultured for 5 days. Representative images of day 5 spheroid cultures are shown in Fig. 3a and results from bioluminescence measurements (day 5) in Fig. 3b. After normalizing bioluminescence measurements to that of non-irradiated controls, significantly increased relative bioluminescence was observed at 4 and 6 Gy doses. Taken together, these results demonstrated that WR-1065 provided radioprotection and validates the bioluminescent SFA for identifying novel radioprotectors.

### Application of Bioluminescent SFA for identifying radiosensitizers

**Figure 4:**
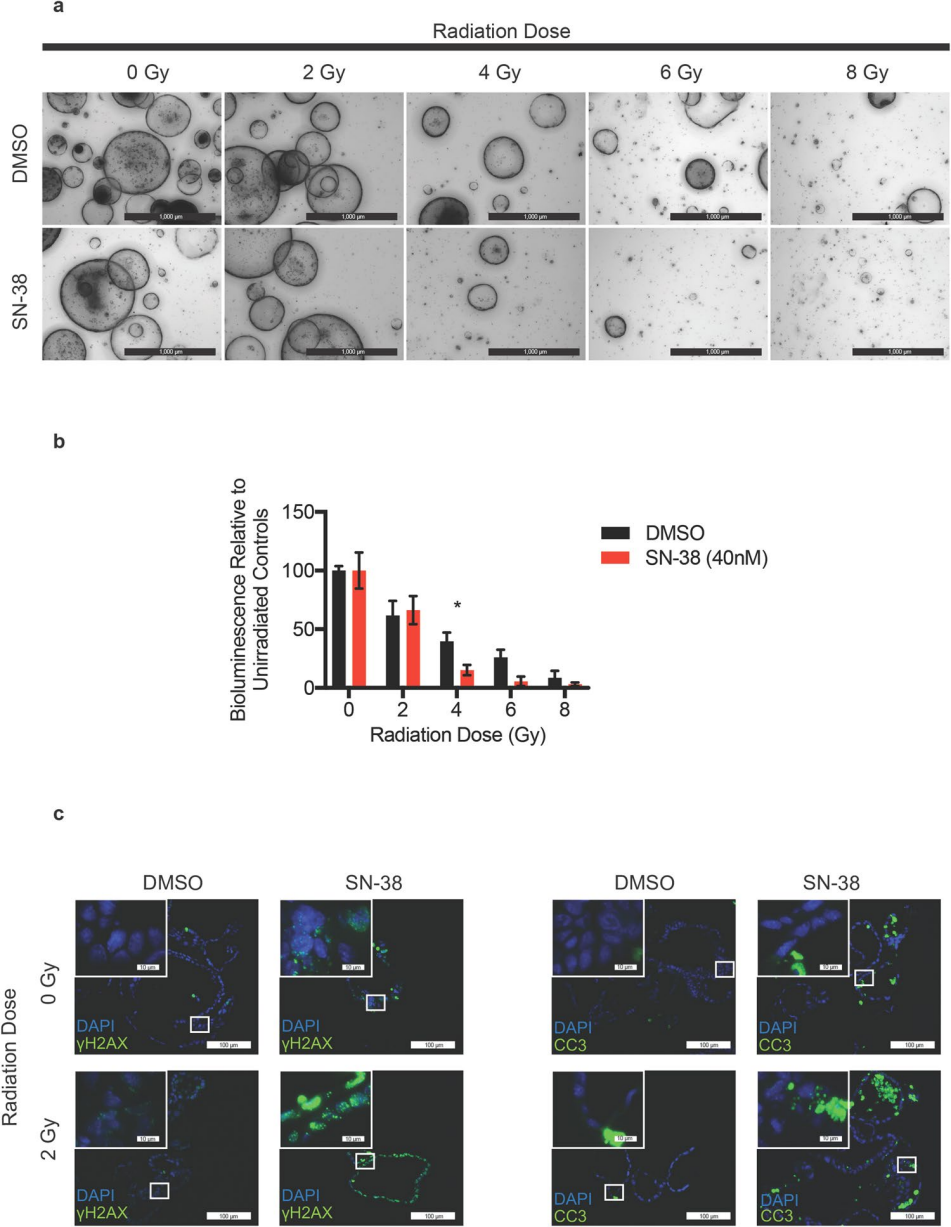
Spheroid Formation Assay for identifying radiosensitizers *in vitro*. Stem cell-enriched epithelial spheroid cultures were incubated in the presence of vehicle (DMSO) or 40 nM SN-38 for 24 h and then either mock irradiated or exposed to increasing doses of ionizing radiation. Spheroids were immediately dissociated to single cells and 3,000 cells were plated per well in triplicate and cultured for 5 days. Representative bright field images (panel a) (Scale bars, 1000 μm) and corresponding bioluminescence measurements (panel b) are shown for day 8 samples. *P < 0.05 by two-tailed, Student’s t test (N = 3 per group). Error bars are ± SEM. Stem cell-enriched epithelial spheroid cultures were incubated in the presence of DMSO or 40 nM SN-38 for 24 h and then either mock irradiated or exposed to 2 Gy IR. Spheroids were harvested 3 h later and stained for γH2AX or cleaved caspase-3. Representative images are shown. Scale bars, 100 uM and 10 uM (inset) (Panel c).

The SFA was also tested for its ability to detect radiosensitizers. Irinotecan, a topoisomerase I inhibitor used clinically to treat colorectal cancer^14^, is known to radiosensitize cells and cause mucositis and diarrhea in both patients and mice. We tested the capacity of the SFA to correctly identify radiosensitizers by treating cells with SN-38, the active metabolite of irinotecan. Cells were plated at a density of 5,000 cells per well and allowed to grow for 5 days. Stem cell enriched-epithelial spheroid cultures were incubated in the presence of DMSO (vehicle) or 40 nM SN-38 for 24 h, and then either mock irradiated or exposed to increasing doses of ionizing radiation. Spheroids were then immediately dissociated to single cells and 3,000 cells were plated per well in triplicate and cultured for 8 days. As expected, fewer spheroids were visualized in SN-38 treated cultures relative to vehicle-treated cultures in a dose dependent manner (Fig. 4a). Concordantly, significantly less relative bioluminescence was measured in SN-38 treated cultures relative to vehicle-treated cultures at 4 Gy of IR (Fig. 4b), demonstrating that irinotecan was functioning as a radiosensitizer in this assay.

Spheroids were also analyzed for the presence of DNA double strand breaks (DSBs) and apoptosis following SN-38 and IR treatments. Spheroid cultures were incubated in the presence of DMSO or 40 nM SN-38 for 24 h and then either mock irradiated or exposed to 2 Gy IR. The culture media was immediately replaced with fresh media lacking SN-38 and spheroids were cultured for an additional 3 h. Spheroids were then processed and stained for antibodies specific for either γH2AX (gamma H2A histone family, member X) to assess DNA DSBs or for cleaved caspase- 3 to assess apoptosis (Fig. 4c). SN-38 as a single agent induced more DNA DSBs and apoptotic cell death than did exposure to 2 Gy IR alone. However, the combination of SN-38 and IR induced substantially more DNA DSBs and apoptosis than either agent alone, supporting a role for SN-38 as a radiosensitizer.

## Discussion

The development of a rapid, high throughput assay that can be performed *in vitro* to identify novel GI radioprotectors and radiosensitizers has the potential to greatly impact the field of oncology. This is due to the fact that one of the major side effects of chemotherapy and radiation therapy is toxicity to the small intestine. The current gold standard Withers and Elkind assay is costly, low throughput, and time consuming^7^, and currently impossible to do in human tissue, which has limited they discovery of novel modulators of radiation responses in normal tissue. Here we describe a novel *in vitro* assay for evaluating intestinal radioprotectors and radiosensitizers that overcomes these previous limitations. Our assay is rapid, high throughput, closely approximates more time-consuming methods, and could be easily extended to human tissues as needed.

Our assay employs three-dimensional cultures that are enriched for SI stem cells and grow as multicellular spheroids that may better approximate *in vivo* biology than standard 2D cultures^10^. In addition, we have created intestinal stem cell lines with a luciferase reporter that acts as a surrogate for live spheroids after a cytotoxic insult since luciferase requires ATP for its enzymatic activity^15^. This facile system will enable screens using unbiased small molecule libraries^16^ or CRISPR/Cas9 systems^17^ that could quickly identify targets that radiosensitize or radioprotect the intestine. Finally, although the SFA described herein employs stem cell-enriched epithelial spheroid cultures derived from the small intestine, it should be feasible with any normal or malignant tissues that grows as multicellular spheroids *ex vivo*^18^.

## Data Availability

Materials, data and associated protocols available upon request.
